# Clinical Integration of Community Health Workers to Reduce Health Inequities in Overburdened and Under-Resourced Populations

**DOI:** 10.1089/pop.2021.0376

**Published:** 2022-04-19

**Authors:** Stacy Ignoffo, Helen Margellos-Anast, Melinda Banks, Rachel Morris, Kim Jay

**Affiliations:** Sinai Urban Health Institute, Sinai Chicago, Chicago, Illinois, USA.

**Keywords:** care management, community health, health disparities, health equity

## Abstract

The COVID-19 response has resulted in broader awareness of health inequities across the United States and their impact on overburdened and under-resourced communities. Investing in and more effectively integrating community health workers (CHWs) into health care delivery been prioritized in the COVID-19 response given the importance of trust and community connection to move people toward behavior change during times of uncertainty. CHWs serve as liaisons and connectors between patients, communities, and health/social care systems, providing culturally appropriate education and addressing complex social needs within the individual and community context. Given the pervasive health inequities that continue to persist despite decades of efforts to curb them, health care systems should reimagine current care delivery models to fully integrate CHWs into care teams. However, barriers exist to effectively deploying CHWs in health care systems. Through 20 years of experience developing, implementing, evaluating, and scaling CHW interventions, Sinai Urban Health Institute has learned valuable lessons in overcoming the common barriers to true and effective CHW integration. Organizations that approach CHW program implementation with a deliberate focus on recruitment and training and career pipelines/pathways, and adequately prepare their organization for CHWs will realize the benefits this unique workforce has to offer. Our experiences have demonstrated that if you hire the right people, train them effectively, and provide appropriate supervision, CHWs are transformative to health care delivery. We discuss our solutions in these areas within the context of integrating CHWs into our health care system to work with our most medically and socially complex patients.

This commentary highlights 1 safety net health system's 20+ year journey to develop, train, support, and sustain community health workers (CHWs) as critical resources supporting value-based care (VBC) health care delivery and health equity for overburdened and under-resourced (OUR) communities. The authors present the case and lessons in the context of the COVID-19 response, which has amplified awareness of health inequities and their impact on OUR communities across the United States, and has led to enhanced federal investments toward building the CHW workforce.

This investment is demonstrated by the Biden Administration's plans to more than triple the number of CHWs who are part of a national effort to improve chronic conditions, and Partners In Health calling for a national community-based Public Health Job Corps to add 540,000 CHWs for 3 years^1^ (to the estimated 58,670 CHWs in the United States as of May 2020).^2^ Given recent trends, it is reasonable to project that this number has increased substantially and will continue to. As of May 2021, ∼18 states have programs that support the certification and reimbursement of CHWs, with ∼25 state programs under development/consideration.^3^

Those familiar with the impact CHWs have in improving health outcomes have advocated for the expansion of the workforce as central to achieving health equity for some time. It is not surprising that a hyperlocal personalized response centered on CHWs (or similar workforces including resource coordinators, vaccine ambassadors, and community contact tracers) proved invaluable to the COVID-19 response. Movement toward recognizing the CHW workforce through mechanisms such as state-wide certification and reimbursement, combined with this national “push,” has the potential to transform the health of OUR populations. Herein these statements are supported within the context of existing health care delivery models.

Over the past 15 years, VBC programs have evolved from mostly hospital-focused measures (eg, readmissions, hospital-acquired infections) to full-risk capitation models and variations in between. Attempting to bridge the gap between patients and health care, providers have deployed armies of clinically trained care managers to coordinate medical treatment, complete health assessments, develop care plans, and monitor patients' progress. Although licensed care managers are valuable in augmenting clinical support, their primary scope of work typically does not include addressing the complex health-related social needs within the individual and community context of the patients, nor do they function as liaisons between the health care system and community. A modified model is needed, given that health inequities persist^4^ despite these efforts.

An alternative care model that integrates CHWs into care teams better supports providers' and payers' VBC objectives. Strategic partnerships between CHWs and traditional members of care teams support the formation of *a health care workforce working for, by, and with the community.* CHWs address barriers related to social determinants of health; serve as connectors between patients/communities and health/social service providers; and increase patient knowledge and self-sufficiency through outreach, navigation, culturally appropriate education, informal counseling, social support, and advocacy.^5,6^

They are unique in that they reflect the communities served by the health system, can relate to patients in a personal way that leads to trusting relationships, focus on facilitating connections between organizations/systems, and actively empower patients toward decisions to enhance their well-being. CHW interventions have been widely studied and recommended based on evidence of improved health outcomes in diabetes and cardiovascular management; diabetes prevention; increased screening for breast, cervical, and colorectal cancers, among other areas; and cost effectiveness,^7^ with return on investment of 3:1 for Medicaid enrollees with unmet needs.^8^

Health care settings are increasingly hiring and integrating CHWs into care teams. Not only do CHWs improve health outcomes, improve patient experience, and reduce utilization, but given the intent that CHW programs hire directly from the communities they serve, health care organizations have a hand in creating local economic opportunities and supporting equity-related efforts. However, it is critical to acknowledge that barriers exist to *effectively* integrating CHWs into health care systems.

Integrating a successful CHW workforce into health care settings requires intentionality to develop effective recruitment and training models and career pipelines/pathways, and to prepare the organization for CHWs. Sinai Urban Health Institute's (SUHI) experiences >2 decades demonstrate that, if you hire the right people, train them effectively, and provide appropriate supervision, CHWs are transformative to health care delivery. The following case study highlights how SUHI addresses the most common barriers.

As the community-engaged research arm of Sinai Chicago (SC), Illinois' largest private safety net health care system, SUHI was an early adopter of the CHW model—developing, implementing, evaluating, and scaling effective CHW models for >20 years. In 2017, SUHI launched the Center for CHW Research, Outcomes, and Workforce Development (CROWD), a CHW training and consulting center. CROWD has worked with 56 organizations to integrate the CHW model and trained >1700 CHWs. Evaluation of CROWD's CHW training has yielded positive feedback.

After 2 recent CHW core skills trainings (the foundational training for CHWs), 100% and 97% of attendees, respectively, reported that their confidence in their skills had improved and that they felt confident in applying what they learned to address health inequities in the communities they serve. CROWD is currently working to integrate CHWs into departments within SC including ambulatory care, outpatient surgery, gastrointenstinal, women's health, among others, adding up to 15 CHWs to SUHI's existing 13 in support of SC patients and the broader community.

SUHI's early work included CHW-led home-based chronic disease management interventions for asthma and diabetes, and community-based outreach, education, and navigation for breast cancer screening. These interventions demonstrated 70%–80% reductions in asthma-related emergency department visits and hospitalizations^9^; clinically significant decreases in hemoglobin A1C levels and an increase in the percentage of participants with controlled diabetes; and 28,000 individuals being educated on the importance of breast cancer screening and navigated to 5800 mammograms.

To more holistically address the substantial needs of SC patients/communities, SUHI expanded outreach and navigation to include cervical and colorectal cancer screenings, launched a CHW-led National Diabetes Prevention Program, and developed a complex care intervention, the *CHW Support Program*. The latter is a collaboration with SC's social work department to assess readmission risk and refer high-risk patients to CHWs.

Through social and health risk assessments (eg, housing, food and financial security, transportation), CHWs connect patients to resources, make appointments, educate patients on their discharge instructions, and tailor disease and health education, all while communicating with clinical teams and ensuring proper follow-up. The *CHW Support Program* is SC's first example of true intentional integration of CHWs into care teams. Since the program's inception, CHWs have screened >850 patients and connected 80% of screened patients with demonstrated social needs to resources to meet their needs. Importantly, data suggest that screened patients are ∼35% less likely to be readmitted compared with unscreened patients.

The lessons learned in overcoming the common barriers to effective CHW integration are outlined hereunder.

**Barrier: Recruitment**—Traditional workforce recruitment strategies are often insufficient as standalone methods for CHW recruitment. Although awareness of the CHW role is growing, many potential applicants may not fully understand the responsibilities. Also, barriers to internet or technology may make online applications challenging.

Solutions:

- Implement novel recruitment strategies including CHW hiring information sessions, networking around positions with trusted community leaders/organizations, and engagement of community relations departments.
- Grow a pipeline of job-ready individuals through mentorship, internship, and apprenticeship programs.
- Utilize interviewing techniques that prioritize soft skills and assess core competencies needed in a CHW—the goal is to identify a person who, with appropriate training, will thrive as a CHW.

When hiring CHWs, SUHI hosts prescreening informational sessions, a process that supports the identification of candidates. Sessions provide candidates an opportunity to learn more about the CHW role and assess their comfort and fit with the work. Sessions are held at the hiring institution and/or community-based organizations to ensure accessibility for candidates. At these sessions, experienced CHWs educate about all aspects of the role and observe and engage applicants in the session through posing questions, role plays, and other methods. Interview techniques focus on soft skills—interpersonal characteristics that can demonstrate how potential CHWs will interact with others. Soft skills, combined with effective and comprehensive training, are the keys to success.

**Barrier: Comprehensive training and supports**—CHW training programs are often incomplete or overly standardized and not personalized to the needs of trainees. Valuable lived experience of trainees should be leveraged in the training process. The initial training session is the first step in a process toward ongoing upskilling and growth of the workforce through continued training in classroom and field settings.

Solutions:

- Incorporate adult learning and popular education models into training.
- Provide a multilayered training approach that includes core skills, specialty training, and ongoing support and training opportunities.
- Plan for longer field training and mentoring, including shadowing, observations, and assessments, before CHWs are assigned to independently manage a caseload.

*CHW Support Program* CHWs complete core skills training through CROWD. Core skills training includes competencies that align with the nationally recognized CHW Core Consensus (C3) Project (www.C3project.org), and is developed and offered by an experienced multidisciplinary team of CHWs and content experts. CROWD uses the popular education model to conduct trainings.^10^ This approach relies on active participation from both teachers and learners to bring forth knowledge from all participants. This model enhances engagement and understanding, and provides relevant on-the-job application through discussion and other interactive methods.

In addition, specialty training is provided on assessing/addressing health-related social needs, intervention protocols, and data collection and technology. Before new CHWs are deployed independently, they shadow and receive mentorship from an experienced CHW for several months. Longer term CHWs frequently meet, discuss their work, bring forward difficult cases, discuss solutions and successes, and identify additional training needs. These “learning collaboratives” are critical to the work and continued growth of CHWs.

**Barrier: Supportive and knowledgeable supervision**—As a novel workforce, the CHW role may not yet be well understood. Attention to supervision is thus important in laying the groundwork for a CHW's success.

Solutions:

- Promote experienced CHWs into CHW supervisor roles.
- Provide training for CHW supervisors.
- Conduct weekly debrief sessions for CHWs.

*CHW Support Program* CHWs are supervised by experienced CHWs who have demonstrated strong leadership and team-building skills. They also receive mentorship from active CHWs who have multiple years of experience. SC CHW integration efforts have revealed that clinicians can also provide effective supervision when they understand the CHW role. Backgrounds in social work, care coordination, and/or patient education seem most adept at supervising the workforce. Regardless of the previous experience of CHW supervisors, formal training is recommended; the goal of which is to acclimate supervisors to the diversity and capacity of the CHW role and how to effectively manage, mentor, and support CHWs.

**Barrier: Career progression and pathways**—Career ladders within the CHW profession are not well defined. Many speak of the CHW role as an entry way into health care or public health, however, the CHW role itself should be considered a valuable career option. To that end, career pathways within the CHW profession need to be formally considered and established.

Solutions:

- Build CHW career ladders to allow for growth and retention within organizations.
- Offer cross-training opportunities for CHWs to work within multiple program areas (eg, asthma and diabetes management).
- Educate hiring institutions about the profession so they understand the role, effective hiring practices, and career pathways.

Over time, SUHI/CROWD developed well-outlined career pathways for CHWs that include 3 levels for CHWs providing direct service to SC patients/communities. Pathways were also developed for those who have the desire and skills to move into supervision, training, or program coordination roles. The *CHW Support Program* benefits from a robust team that includes individuals newer to the field, experienced CHWs, CHW supervisors, and a CHW program coordinator.

Although the CHW position has not historically been well understood, integrated, and/or supported within the health system, the tide is turning and more organizations are beginning to understand the benefits of including CHWs in their care teams. Avoiding the common pitfalls discussed will lead organizations to fully realize these benefits for themselves and patients alike.

## Authors' Contribution

The authors of this article have all contributed significantly to the preparation of this commentary.
